# RNA-based scaffolds for bone regeneration: application and mechanisms of mRNA, miRNA and siRNA

**DOI:** 10.7150/thno.42640

**Published:** 2020-02-10

**Authors:** Qiuping Leng, Lini Chen, Yonggang Lv

**Affiliations:** 1 Key Laboratory of Biorheological Science and Technology (Chongqing University), Ministry of Education, Bioengineering College, Chongqing University, Chongqing 400044, P. R. China; 2 Mechanobiology and Regenerative Medicine Laboratory, Bioengineering College, Chongqing University, Chongqing 400044, P. R. China

**Keywords:** RNA-based therapy, mRNA, siRNA, miRNA, scaffold, nonviral vector, bone regeneration, delivery

## Abstract

Globally, more than 1.5 million patients undergo bone graft surgeries annually, and the development of biomaterial scaffolds that mimic natural bone for bone grafting remains a tremendous challenge. In recent decades, due to the improved understanding of the mechanisms of bone remodeling and the rapid development of gene therapy, RNA (including messenger RNA (mRNA), microRNA (miRNA), and short interfering RNA (siRNA)) has attracted increased attention as a new tool for bone tissue engineering due to its unique nature and great potential to cure bone defects. Different types of RNA play roles *via* a variety of mechanisms in bone-related cells *in vivo* as well as after synthesis *in vitro*. In addition, RNAs are delivered to injured sites by loading into scaffolds or systemic administration after combination with vectors for bone tissue engineering. However, the challenge of effectively and stably delivering RNA into local tissue remains to be solved. This review describes the mechanisms of the three types of RNAs and the application of the relevant types of RNA delivery vectors and scaffolds in bone regeneration. The improvements in their development are also discussed.

## Introduction

Bone is a complex and dynamic tissue endowed with excellent regeneration capacity. However, large or unstable fractures are disadvantageous to successful healing and require additional treatments before they regenerate 1. Tissue engineering uses specific three-dimensional (3D) structural biomaterials (generally known as “bone scaffolds”) to replace and restore defects 2, 3 and to overcome the limitations of autogenous and allogenous grafts. Currently, by means of processing natural and synthetic biomaterials, scaffolds can be easily obtained and designed with different geometric shapes for bone defect healing 4. In addition, to enhance bone regeneration, cells or drugs are added into scaffolds and are delivered to bone defect sites together. Excitingly, over the last few decades, RNA-based therapies have been increasingly used to repair bone defects, as the understanding of molecular mechanisms, such as gene regulation networks that include molecular triggers, signaling molecules, and transcriptional regulators etc. 5 and cellular processes that include endochondral ossification and intramembranous bone formation 6 has gradually increased.

**Figure 1:**
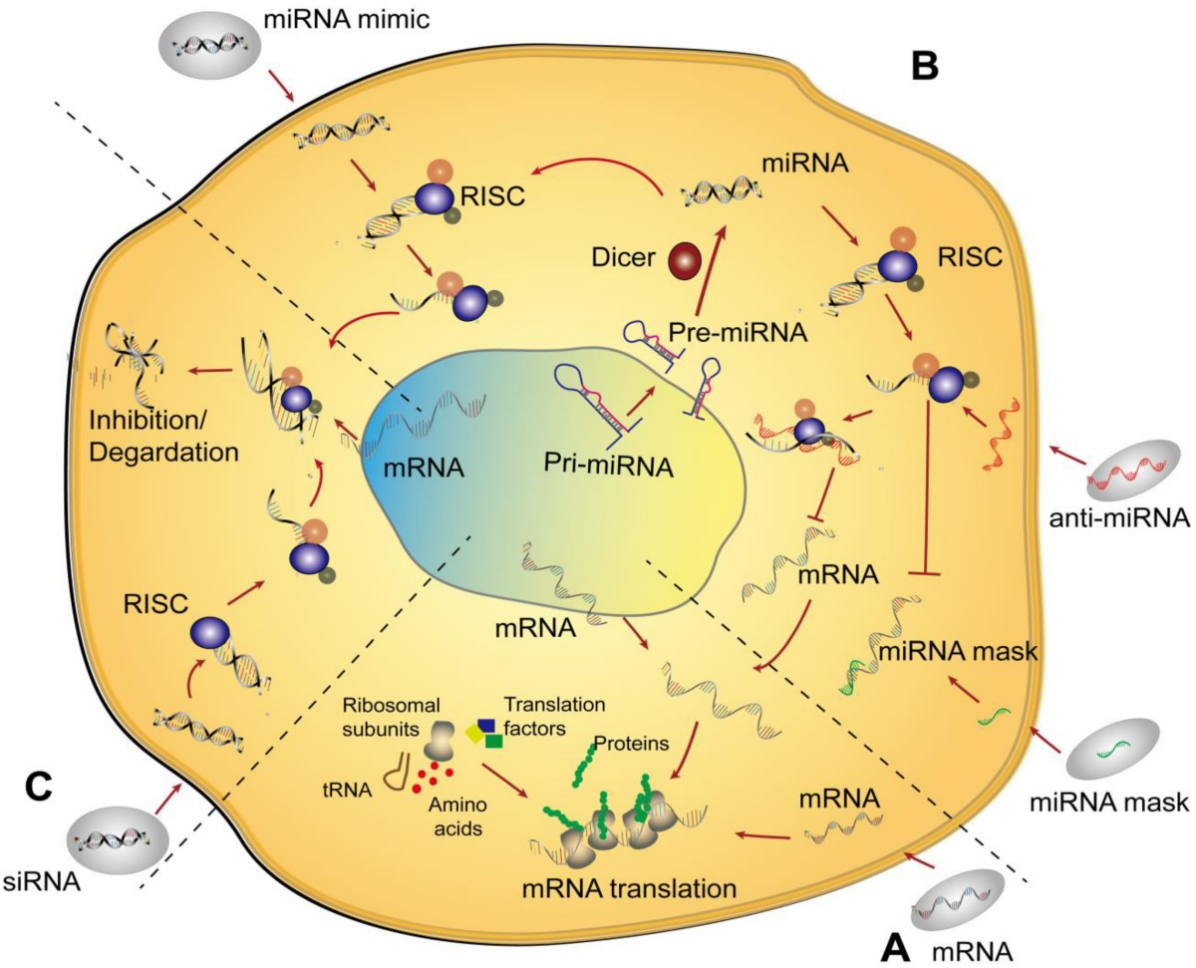
** Schematic of the mechanisms of nonviral RNA therapy in cells. (A)** An mRNA is delivered into the cytoplasm and then translated into the corresponding protein *via* endogenous mRNA translation. **(B)** Three typical types of miRNAs are delivered to cells. An miRNA mimic enters the cell and acts similar to a normal intracellular miRNA. Anti-miRNA, which is an antisense strand of miRNA, combines with miRISC after delivery to cells resulting in the silencing of complementary mature miRNA and further increases in the translation of the corresponding mRNA. An miRNA mask can bind to the 3´ UTR of an mRNA to prevent the function of miRNA. **(C)** Delivery of siRNA utilizes a similar pathway as that of an miRNA mimic.** Abbreviations:** pre-miRNA: precursor miRNA; pri-miRNA: primary miRNA; RISC: RNA-induced silencing complex; tRNA: transfer RNA.

A large variety of RNA therapeutics for bone regeneration have been developed based on the diverse family of RNA molecules, including messenger RNA (mRNA) 7, 8, microRNA (miRNA) 9-11, small interfering RNA (siRNA) 12, 13, and long noncoding RNA (lncRNA) 14-16. Different RNA-based therapeutics, when combined with different scaffolds, can enhance bone defect repair at the RNA level through diverse mechanisms. For example, mRNA in the cytoplasm can be directly supplemented through several methods and then translated into additional therapeutic proteins **(Figure 1A)**. miRNA upregulates osteogenic-related genes or downregulates adverse genes at the posttranslational level **(Figure 1B)**. siRNA can be designed to silence a specific gene that inhibits bone formation through a particular endogenous pathway **(Figure 1C)**. The main mechanism of lncRNA involves the indirect regulation of genes through the binding of competing endogenous RNAs (ceRNAs) with miRNAs during osteogenesis 17. However, the use of lncRNAs has been limited to exploration with a small number of transfected cells in bone tissue engineering and is therefore not described and discussed in detail in this review.

As molecular technology, nanotechnology and the use of novel biomaterials have advanced in the last few decades, therapeutic RNAs can be easily synthesized, delivered to bone and modified according to specific needs. Although naked RNA molecules have been used to repair bone-related disease 18, the instability of RNA has hindered its use. Therefore, modification of RNA is beneficial. Generally, two methods has been used to deliver RNA to bone tissue, namely, systemic delivery and local delivery to the bone sites. Two commonly applied methods of systemic delivery are viral vectors and nonviral nanoparticles. Local delivery to the bone defect site primarily utilizes nonviral biocompatible scaffolds, which offer intrinsic advantages but also show some disadvantages. Previous reviews 19-21 have summarized the applications of the loading of different types of RNAs onto/into scaffolds in bone tissue engineering.

However, no study has yet systematically summarized the roles of these RNA-based biomaterials in local delivery. Hence, this review will discuss the mechanisms of the three types of RNAs, namely, mRNA, miRNA, and siRNA, with an emphasis on the classification of these RNA delivery systems based on the different scaffolds used and the description of clinical trials and therapeutic applications of these RNA delivery biomaterials in the field of bone defect repair. In addition, the advantages and limitations of RNA therapies are discussed.

## mRNA-based therapy for bone repair

### mRNA preparation for bone defect repair

mRNA is a type of single-stranded ribonucleic acid that is transcribed from one strand of DNA, which functions as a template, and carries genetic information to direct protein synthesis in eukaryotic cells *in vivo* 22. *In vitro* transcription mRNA (IVT mRNA), rather than endogenous mRNA, has tremendous potential to repair bone defects and represents a new drug class. The use of IVT mRNA avoids the obstruction of the nuclear membrane that occurs due to the transfection of plasmid DNA (pDNA), which is inefficient as a means of gene therapy. mRNA does not have to enter the nucleus to be effective, which not only avoids the barrier posed by the nuclear membrane 23 but also results in higher effectiveness in nonmitotic cells 24. Moreover, there is no risk of genomic integration after introducing mRNA, and mRNA has no immunogenic CpG island motifs, unlike pDNA. These issues remain a major concern for DNA-based gene therapy 25, 26. The excellent properties of mRNA ensure that high-efficiency, controlled and rapid onset of therapeutic proteins expression can be obtained by mRNA-based treatments. Transcription of mRNA *in vitro* requires mimicking of intracellular transcriptional environment. Generally, IVT mRNA is transcribed from a linearized pDNA or a polymerase chain reaction (PCR) template with a bacteriophage promoter, a 5´ untranslated region (UTR), an open reading frame (ORF), a 3´ UTR, and an optional poly[d(A/T)] sequence 23. The instability of mRNA due to its inclusion of hydroxide radicals and surrounding nucleases has evoked some concerns. Undoubtedly, the inclusion of chemically modified nucleotides in the cap structure, 5´ or 3´ UTR, ORF, or other parts of the mRNA offer a desirable solution for ensuring the stability and translatability of an mRNA 7, 27, 28.

### mRNA delivery to bone-related cells

#### Physical methods

In 1969, mRNA was successfully transcribed *in vitro* for the first time 29. This appeared to be useful for application to vaccines development, immunotherapy of cancer, and treatment of various other diseases 30. Delivery of mRNA to the cytoplasm can be achieved by both physical methods (electrotransfection 31, a gene gun 32, or microinjection 33, 34) and chemical methods (cationic polymers, liposomes, liposome nanoparticles, etc.). For example, electroporation is considered to be a highly effective physical method for the direct delivery of mRNA into the cytosol 31, 35, which is achieved *via* applying electrical pulses to the cell membrane so that mRNA can enter the cytoplasm. Nevertheless, until now, few studies have reported the use of these techniques in bone tissue engineering to achieve mRNA delivery due to the high cost and inconvenience of *in vivo* delivery.

#### Chemical methods

Compared with physical methods, the applications of chemical methods have been widely explored in the repair of bone defects 36 due to their low cost, user friendliness, and multifunctionality. Among chemical carriers, cationic polymers, lipoplexes, and lipid nanoparticles have been used mainly in mRNA-based bone tissue engineering. For example, the cationic polymers poly-L-lysine (PLL) and polyethyleneimine (PEI) 7, 37, two typical types of cationic polymers with positive charges, can bind to negatively charged mRNA to form nanoparticles that can be absorbed by cells *via* the endocytosis. In terms of delivery into the cytoplasm, PEI is more efficient than PLL 38 that did not result in detectable translation of mRNA 36. Another important vector is lipoplex nanoparticles (or their derivatives), which have been used for a wide variety of mRNA delivery methods 39-41. After mixing nucleic acids (negative charges) with cationic liposomes (positive charges), these components will spontaneously assemble into multiphase complexes through electrostatic interactions 24. Nanoparticles have assumed various forms, such as polyplexes, lipoplexes, lipid-nanoparticles (LNPs), dendrimers, inorganic nanoparticles, and hybrid nanoparticles 41. In a recent study, researchers developed improved biodegradable lipid nanoparticles to deliver mRNA into the liver for CRISPR/Cas9 gene editing, which accurately altered the genetic code of cells with an efficiency as high as 90% 42. In conclusion, lipoplex nanoparticles are the most effective mRNA delivery tools reported to date and may overcome many technical barriers. Other cationic polymers have been discussed in previous reviews 36, 43.

### mRNA delivery to bone defect sites

mRNA possesses superior properties for the preparation of tumor vaccines 44-48, cancer immunotherapy 49, drug delivery 50, skin repair 51, myocardial damage repair 52, and other fields of regenerative medicine. However, it was not until 2015 that the enormous potential of mRNA in bone tissue engineering was discovered by Elangovan et al. 7, who first used mRNA combined with tissue engineering scaffolds to repair bone defects and thereby opened a new avenue for bone regeneration.

#### mRNA modification

Regardless of the field, one of the main limitations of mRNA applications is immunogenicity, as unmodified mRNA is recognized by innate immune sensors that activate genes that inhibit translation 53. To reduce the immunogenicity of mRNA, Elangovan et al. 7 modified the ribonucleic acid triphosphates of the mRNA coding region by replacing guanosine and cytosine with thioglycosylated guanosine and methylated cytosine, and the modified mRNA was called cmRNA. As mentioned before, cmRNA has lower immunogenicity than unmodified or mRNA with 25% modification. Subsequently, a complex of bone morphogenetic protein-2 (BMP-2)-encoding cmRNA and PEI was combined with a collagen scaffold for the repair of a rat skull defect. As expected, the cmRNA-modified scaffolds significantly accelerated process of bone regeneration, while pDNA-modified scaffolds had limited effects on bone healing. By using this method, Elangovan et al. 7 perfectly demonstrated the powerful potential of cmRNA-modified scaffolds in bone tissue engineering by demonstrating the efficacy of cmRNA for such application. Based on this study, the Elangovan team members 37 further compared the promotion of regeneration in calvarial defects of rats by cmRNA encoding BMP-2 and cmRNA encoding BMP-9 after electrostatic interaction with the collagen scaffold. The osteoinductive capability of BMP-9-cmRNA was stronger than that of BMP-2-mRNA, which was contrary to common belief that BMP-2 is a useful therapeutic molecule in the clinic 54. Certainly, their results lacked sufficient evidence to completely disprove the effectiveness of BMP-2. Subsequently, Zhang et al. 28 designed a new mRNA that deleted an upstream open-reading frame in the 5′-UTR and an AU-rich tract polyadenylation element in the 3′-UTR. 5-iodo-modified pyrimidine nucleotides were also introduced into the mRNA as a translation initiator for short UTRs (TISU), which show significant advantages in enhancing new bone formation.

#### Vector improvement

The improved cmRNA appeared to have a lower immunogenicity. However, the release duration of mRNA, which is one of the key issues in affecting mRNA applications for bone regeneration, did not seem to be considered in previous research. Improving mRNA stability and prolonging its effects, even when it shows low immunogenicity, are still urgently needed. The Plank laboratory has focused on the research of advanced magnetic liposome transfection vectors 55-58. On the one hand, it was assumed that using these advanced vectors with a high transfection efficiency to deliver osteogenic-related mRNA could promote bone formation in bone defect sites. On the other hand, the use of a linearized template resulted in difference in translation efficiencies compared to those of mRNAs with modified ribonucleic acid triphosphates. For example, a NotI-linearized template possessed a high translation efficiency compared to that of the XbaI-analogue reported by Elangovan and coworkers 7. Under double remodeling, lipoplexes and magnetic lipoplexes were used to deliver enhanced-green fluorescent protein (eGFP)-cmRNA to rat adipose mesenchymal stem cells (AMSCs) or bone marrow mesenchymal stem cells (BMSCs) 27. The results indicated the higher efficiency of the magnetic lipoplexes, which produced 1.5-fold increased fluorescence. The testing of alkaline phosphatase (ALP) activity and Alizarin Red staining (RAS) also showed that magnetofection was able to deliver human BMP-2 (hBMP-2)-cmRNA into the cytoplasm more efficiently in both AMSCs and BMSCs than lipofection. To detect the capacity for enhancing bone regeneration of these complexes *in vivo*, hBMP-2-cmRNAs/lipids were combined with fibrin gel scaffolds and implanted into noncritical femoral bone defects in rats. The hBMP-2-cmRNA/scaffolds were able to accelerate bone formation according to μ-computed tomography images and histomorphometric analysis after 2 weeks.

### mRNA-based scaffolds improved bone regeneration

In tissue engineering, the stability and sustained release of loaded molecules are important when the scaffold is used to deliver small molecules. After initially confirming the superior potential of mRNA for bone tissue engineering, it was necessary to determine how to prepare stable and sustainable scaffolds to deliver mRNA. The scaffold parameters (such as biocompatibility, biodegradability, chemical composition, internal structure, pore size, and mechanical properties) should be designed to promote bone remodeling processes 58. There are two main methods of gene transfer used for tissue regeneration. Mesenchymal stem cells (MSCs) are transfected with nucleotide molecules *in vitro* and subsequently transplanted into defect sites. Osteogenic gene-modified scaffolds are directly transplanted into defect sites to recruit MSCs *in vivo*. However, the latter method is more commonly used for mRNA-based therapy for bone regeneration **(Table 1)**. This section describes two typically mRNA-based scaffolds (fibrin gel scaffold and collagen scaffold) and discusses some extra-chemical components, such as calcium phosphate, that could be added to the scaffolds to enhance the formation of bone.

In addition to hydroxyapatite (HA), collagen accounts for a majority of bone tissue and has a complex structure 1. Native collagen occurs in two forms: as a swollen hydrogel or as sparse fiber in a lattice-like organization. Collagen type I, as a raw material, is the type most commonly used 59 to fabricate scaffolds. For nonload-bearing bone defects such as those of the skull, collagen-like scaffolds are applied to simulate the composition of the original bone tissue because of their limited mechanical properties. Badieyan et al. 60 designed vacuum-dried collagen sponge scaffolds preloaded with complexes of cmRNA and lipids that could be associated with the scaffold through weak interactions to ensure a prolonged and sustained release for efficient transfection. In their study, after vacuum drying, the collagen sponge scaffolds possessed a closed structure that entrapped the cmRNA/lipids. Subsequently, the RNA complexes were released from the scaffolds during the gradual degradation of the collagen sponge scaffolds. The research results showed that the scaffolds could provide steady-state protein production for 11 days. In addition, Zhang et al. 28 prolonged the repair time *in vivo* in a similar scenario, which produced ameliorative repair effects for 8 weeks in critical-sized femoral defects in rats.

The stability and sustained release of such scaffolds also evoked the interest of Utzinger et al. 61 and Balmayor et al. 62. Utzinger et al. 61 employed a scaffold that consists of calcium phosphate cement (CPC) wrapped around precoated erythropoietin (EPO)-cmRNA complexes composed of cmRNA and PLGA-based microparticles. The PLGA microparticles rapidly degrade to allow the ingrowth of cells. At the same time, the enhanced degradation produced the steady release of cmRNA complexes. Balmayor et al. 62 compared the rates of molecular release by fibrin gel and micro-macro biphasic calcium phosphate (MBCP) granules in a 3D culture environment after loading them with hBMP-2 cmRNA. First, compared to 2D cultures, the 3D fibrin gel and MBCP granules produced superior gene expression. Second, MBCP played a role in the fast release of cmRNA and decreased gene transfection efficiency, whereas the fibrin gel produced increased cellular internalization. Although these materials were developed for clinically employed materials, further modification of materials should be adopted for mRNA delivery well.

## miRNA-based therapy for bone regeneration

### miRNA in biology

Since lin-4 and lin-7, two small noncoding RNAs, were discovered in succession approximately 20 years ago, thousands of miRNAs have been found to play important regulatory roles in organisms 64. The miRNA is a short, approximately 22-ribonucleotide-long noncoding RNA that exerts vital regulatory functions at the posttranscriptional level of gene expression in multiple organisms *via* activating specific signaling pathways as an endogenous RNA 65. Classically, the journey of miRNAs begins in the nucleus, where they are transcribed into long primary miRNAs (pri-miRNAs) with cervical-loop structures by RNA polymerase II. Afterwards, pri-miRNAs are recognized and processed by Drosha (a member of the RNase III enzyme family) into precursor miRNAs (pre-miRNAs) with a shorter nucleotide length 66. Subsequently, the pre-miRNAs are transported from the nucleus to the cytoplasm through the nuclear pore and processed by Dicer (another member of the RNase III enzyme family) into mature miRNAs 67. In general, miRNA functions *via* combining with the RNA-induced silencing complex (RISC) 68 to form a new silencing complex, termed miRISC. The miRISC recognizes and selectively targets certain areas of the 3′ UTR (or 5′ UTR) of mRNA, with which miRNA is complementary. It should be noted that pairing of miRISC and mRNA is not completely strict, and miRISC does not always target only one mRNA 69. mRNA is not specifically regulated by miRISC 70, which reflects the numerous powerful gene regulatory networks that function in organisms. These networks are gradually being revealed.

Therefore, in each stage of the formation and functioning of mature miRNAs, the corresponding miRNA could be designed to regulate genes. On the one hand, to upregulate the target genes of miRNAs, antisense strands of miRNAs 71, 72, termed anti-miRNA, are mostly designed to combine with miRISC, resulting in the silencing of the complementary mature miRNA (or affecting the maturation of the miRNA). miRNA masks 73 are designed to recognize and coat the 3′ UTR (or 5′ UTR) of an mRNA to block the binding of the miRNA and mRNA, consequently resulting in an increase mRNA and protein expression. On the other hand, to downregulate a target gene, molecules that mimic the design of endogenous miRNAs, known as miR-mimics, can be used to degrade an mRNA or inhibit protein translation, which leads to a decrease in the target protein. In this section, the roles of miRNA in the field of bone defect therapy are described with a focus on the methods of miRNA delivery.

### Role of miRNAs in bone defect repair

**Figure 2:**
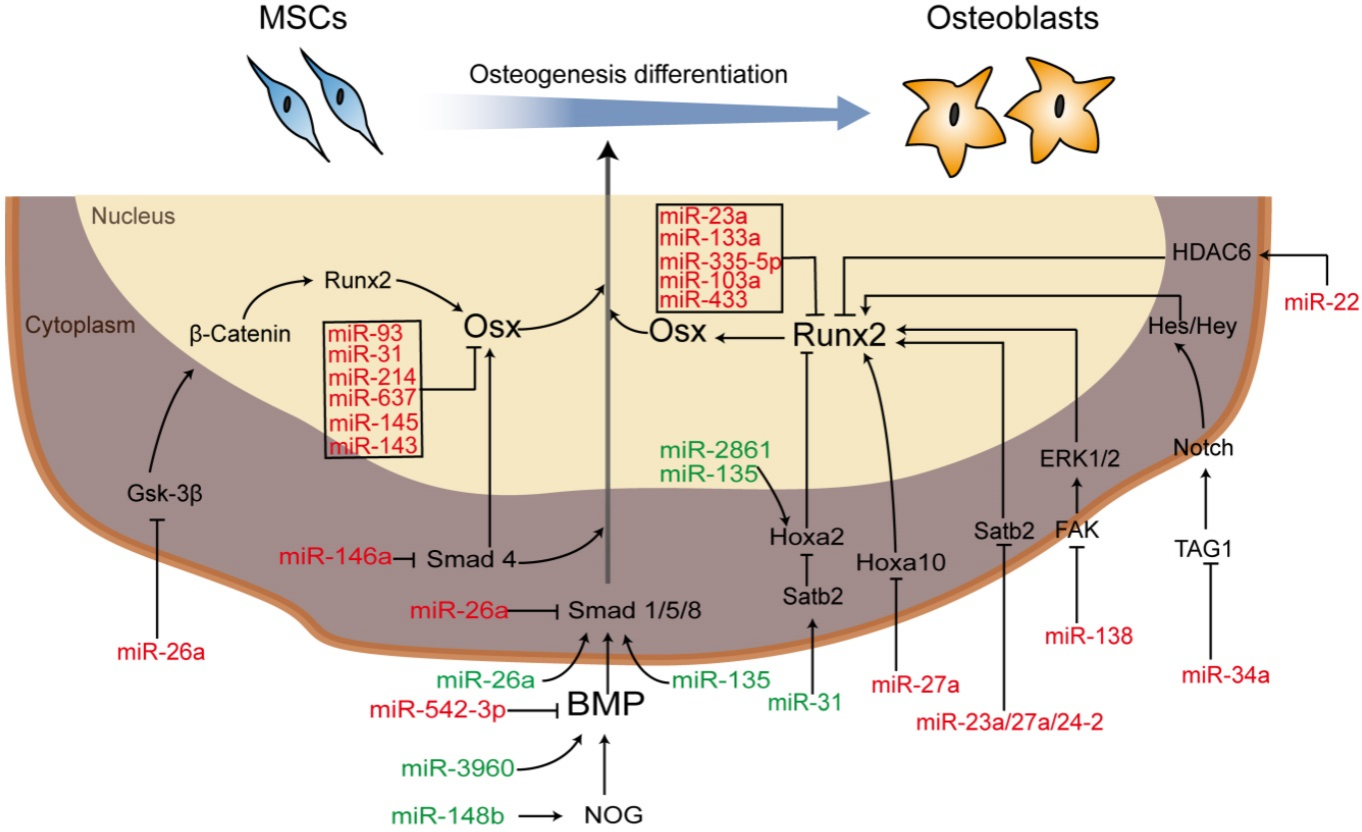
Schematic diagram of miRNA-regulated osteogenic differentiation *via* miRNA scaffold-based therapy. Green indicates upregulation and red indicates downregulation.** Abbreviations:** BMP: bone morphogenetic protein; ERK: extracellular regulated protein kinases; FAK: focal adhesion kinase; Gsk-3β: glycogen synthase kinase; HDAC6: histone deacetylase 6; Hes: hairy enhancer of split; Hey: Hes-related with YRPW motif; Hoxa 10/2: homeobox a10/2; NOG: noggin; Osx: osterix; PI3K/AKT: phosphatidylinositol 3-kinase/protein kinase B; Runx2: runt-related transcription factor 2; Satb2: special AT-rich sequence-binding protein 1; Smad: drosophila mothers against decapentaplegic; TAG1: transient axonal glycoprotein 1.

MSCs, one of the most important seed cells for tissue engineering and regeneration, can be either preloaded onto/into the scaffolds after being modified with RNAs *in vitro* and then implanted into defect or be recruited to RNA-modified scaffolds implanted into defects *in vivo*. Moreover, important classical signaling pathways involved in regulating the osteogenic differentiation of MSCs, such as the BMP signaling pathway and the Wnt/β‐catenin signaling pathway 21, involve several members that can be regulated and influenced by each other or miRNAs as part of complex regulatory networks 74. For example, in the BMP signaling pathway, NOG, the noggin protein gene, prevents the combination of BMP and its receptor to suppress osteogenic differentiation. miR-148b 75, 76 can target NOG to enhance the expression of BMP, while miR-146a 77, 78 binds with Smad4 (phosphorylated by complexes of BMP and its receptor (BMPIA, BPMLB or ALK2)) to suppress osteogenic differentiation. Therefore, an increase in Smad4 is produced by anti-miR-146a. miR-542-3p is a direct target of BMP-7 and function as an inhibitor 79. In the Wnt/β-catenin signaling pathway, runt-related transcription factor 2 (Runx2), also known as core-binding factor A1 (Cbfa1), belongs to the Runx family and plays a role in the BMP signaling pathway as a master osteoblast transcription factor that can be regulated either directly or indirectly. For instance, miR-23a, miR-204 80, miR-103a 81, and miR-467g 82 can directly target Runx2, leading to the repression of Runx2, while miR-135 targets Hoxa2, a negative regulator of Runx2, resulting in the enhancement of osteoblast differentiation 83. Additional miRNA regulators are described in **Figure 2,** and the regulatory effects of miRNAs on bone development *via* different processes are briefly summarized.

Although cell phenotype development requires the regulation of multiple miRNAs, some miRNAs have an opposite effect on the regulation of osteogenic differentiation in different cell types. miR-26a inhibited osteogenic differentiation of human adipose-derived stem cells (hASCs) *via* targeting Smad1 84, while Trompeter et al. 85 showed that miR-26a accelerated osteogenic differentiation in unrestricted somatic stem cells from human cord blood. Chen et al. 71 reported that miR-34a inhibited the osteogenic differentiation of hMSCs and that anti-miR-34a was used to accelerate osteogenic differentiation *in vivo* in mice. However, Liu et al. 10 showed that miR-34a promoted bone regeneration by MSCs in rats *in vivo*. These heterogeneous functions have not yet been fully explored at the same time.

### miRNA delivery to bone-related cells

miRNAs (negatively charged) have been delivered to bone-related cells after binding with vectors (positively charged), such as cationic polymers and cationic liposomes. miRNAs can also be delivered *via* lipid-based vectors, polymer-based vectors (PLAG NPs), or inorganic-based vectors (bioactive glass nanoclusters or nanohydroxyapatite). Studies have shown that the size of the nucleic acid and the physical properties of the vector itself affect the efficiency of transfection. PEI 86, as a cationic polymer, is commonly used to deliver miRNA to the cytoplasm, while lipids and liposome analogues are most commonly applied to deliver miRNA to bone-related cells *in vitro*/*vivo*. These vectors have been studied in depth and are widely commercially available, including Oligofectamine 87, Lipofectamine 2000 81, 88, 89, and Dharmafect 1/2/3/4 90, 91. In addition, to achieve optimal delivery efficiency, Qureshi 92 and Moncal 93 used silver NPs and therapeutic miRNA molecules to form photoactivated silver NPs that were combined by robust thiolate bonding and could be dissociated under a range of UV wavelengths from 350 to 450 nm, resulting in the controlled release of miRNA molecules. Increasing numbers of improved vectors have been exploited for the delivery of miRNA to cells, albeit with limited success in clinical trials, making further studies necessary.

### miRNA-based scaffolds in bone defect sites

**Figure 3:**
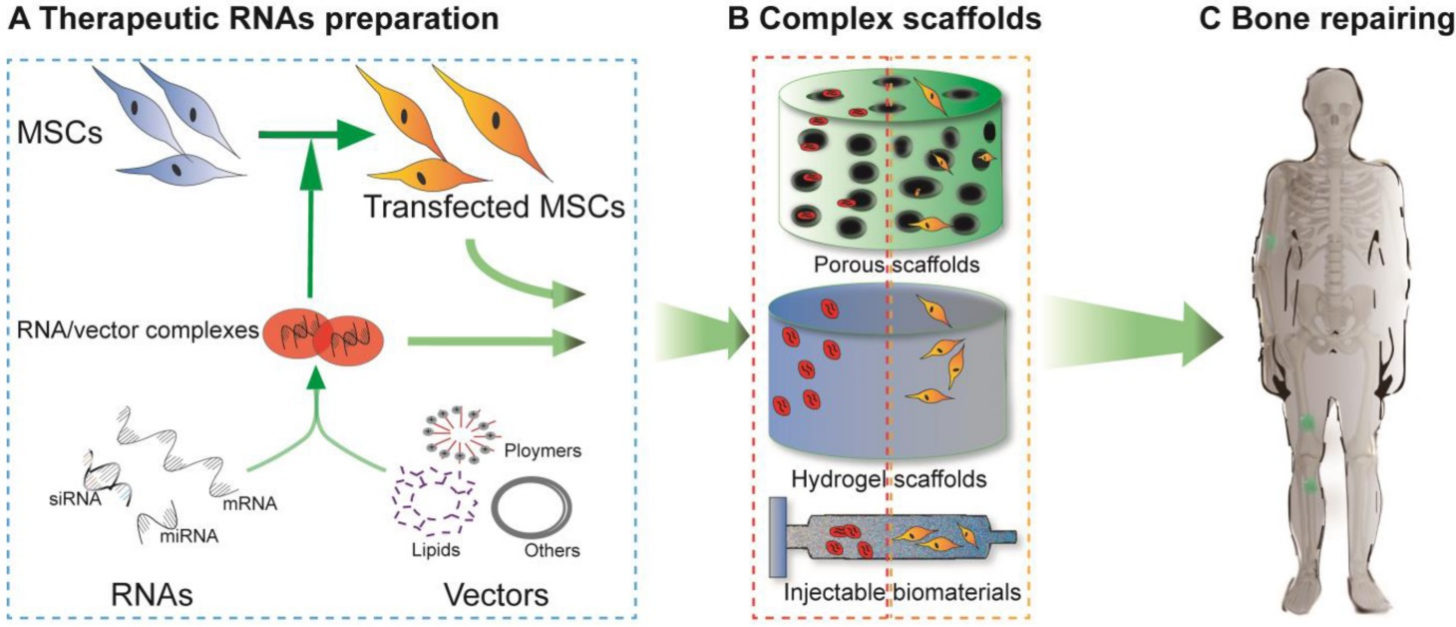
Schematic diagram of RNA-based scaffolds used for bone regeneration. For delivery of nucleic acid molecules, different RNAs were combined with vectors (such as lipids, polymers or others) with or without transfecting MSCs **(A)**. The cell-free scaffolds shown in the red dashed rectangle **(B)** and the cell-loaded scaffolds shown in the orange dashed rectangle **(B)** were designed (*in vitro*) to repair bone defects *in vivo* **(C)**.

Although nano- and microparticles have been synthesized to systematically load miRNAs, these particles are rapidly cleared away from the desired sites after injection *in vivo* due to their small size and discontinuous release. Hence, collagen scaffolds, solid porous scaffolds and hydrogel scaffolds have been developed to release miRNA locally to surrounding cells as they can provide a platform for cell migration, adhesion and differentiation. Therefore, the miRNA-scaffold method is a better method, which has been proven by several studies. Unlike cell-free scaffolds, which are mostly utilized to deliver mRNAs in mRNA therapy, cell-free and cell-mediated scaffolds have both been applied to miRNA delivery. Cell-free scaffold-loaded miRNA/vector complexes are implanted into bone defect sites, termed “*in situ* delivery”. In contrast, for *ex situ* delivery, rat/human BMSCs or AMSCs are transfected with therapeutic miRNAs *in vitro*, loaded onto scaffolds, and ultimately implanted into the damaged areas **(Figure 3)**.

A variety of scaffolds have been used to facilitate the delivery of miRNA. However, some types of scaffolds loaded with nonviral vectors or cells are more commonly applied in bone regeneration, including hydrogel-based scaffolds, HA scaffolds and collagen-based scaffolds. These scaffolds are summarized in **Table 2,** and their applications are reviewed in more detail.

#### Hydrogel scaffolds

Hydrogels are highly hydrophilic materials and ideal for clinical application 94. Injectable hydrogels can be used for many types of geometrical deformities and are more advantageous compared with prefabricated scaffolds 95. According to previous studies, many types of hydrogels can encapsulate miRNA-processed cells for local delivery with high viability 86, 96, 97, and those processed cells are designed to promote bone formation. Nguyen et al. 86 proved the ability of encapsulated miR-20a to process human MSCs (hMSCs) within polyethylene glycol (PEG) hydrogels. These hMSCs (posttransfection)-loaded PEG hydrogel scaffolds were applied to the repair of critical-sized calvarial defects in rats 13, resulting in good bone repair. Instead of using conventional biomaterial vectors, Xue et al. 97 used positively charged bioactive glass nanoclusters (BGNCs) with ameliorative ultra-large mesopores as ideal miRNA vectors. The BGNCs possess gene activation properties when they are delivered to bone with miRNA. Similar to that observed for cationic polymers, there is a strong electrostatic interaction between the negatively charged phosphate groups in miRNA and the positively charged calcium ions in BGNCs. In addition, hydrogels can deliver miRNA/vector complexes without cells to bone defects in a controllable way 98, 99. Cell-free scaffolds are the ultimate goal of tissue engineering due to their ease of use, lower costs and lack of complications in clinical regeneration 100. A two-stage release system was designed to prolong the expression of miR-26a by Zhang et al. 98. PLGA microspheres, containing complexes of miR-26a/hyperbranched polymer (HP) vectors, were encased in PLLA scaffolds, which ensured sufficient time for the transfection of endogenous cells and resulted in a good repair effect. Similarly, anti-miRNA-138/chitosan (CS) nanoparticle (NP) complexes were encapsulated in β-sodium glycerol phosphate (GP) hydrogel scaffolds for controlled release *in situ* by Wu et al. 99. The effect of the anti-miR-138 CS/GP scaffolds was barely adequate and this idea needs to be further explored.

#### Collagen-based scaffolds

Similar to mRNA-based therapeutics, collagen, as the major organic component of the bone extracellular matrix, plays an important role in the delivery of miRNA for bone regeneration. Conventional collagen scaffolds are prepared using type I collagen, which can be formed into a variety of shapes to treat various defects, such as disciform scaffolds for critical-sized calvarial defect. In addition, collagen scaffold can be generated by 3D printing with collagen as bioink 93. As a novel technology, 3D printing can precisely control the biomechanical properties, macrostructures (such as pore size and shape) and microstructure (such as roughness) of scaffolds 101, 102 to meet patient-specific demands with simplified procedures specially. 3D printed scaffolds were loaded with miRNA-transfected cells and applied to bone defect sites, resulting in successful bone healing 93. This method can also be applied to deliver mRNA and siRNA through the loading of mRNA- or siRNA-treated stem cells. However, few studies have reported the use of cell-free 3D printed scaffold loaded RNAs (regardless of which types of RNAs were used) for the repair of bone defects.

As a derivative of collagen, atelocollagen is obtained by removing the collagen terminal peptide with pepsin digestion. High-purity atelocollagen has decreased antigenicity and increased bioaffinity; therefore, it is considered a good choice for bone regeneration. Yoshizuka (Yoshizuka et al., 2016) 72 demonstrated its outstanding effects in a refractory fracture model.

#### HA-based scaffolds

Based on previous results, HA accounts for 70% of bone composition and provides mechanical support for the body 103. When it is added to hydrogel or collagen, HA enhances the mechanical properties of composite scaffolds. In general, HA alone can form a scaffold 104, and HA and tricalcium phosphate (TCP) can form a composite biomaterial 9, 71, 105. Sometimes, HA, in the form of nano HA (nHA), can form composite scaffolds together with collagen or CS for use in miRNA delivery 106-107. Unlike previous methods that encapsulated miRNAs in liposomes, nHA can directly carry miRNAs in combination with other components to form a composite scaffold. Interestingly, HA-based scaffolds are commonly used to detect ectopic bone formation under the endothelium *in vivo*.

## siRNA-based therapy for bone repair

### siRNAs in biology

Endogenous siRNAs are short (19-30 nucleotides) double-stranded RNAs (dsRNAs) with two nucleotide-overhangs in the 3' UTR 115. Unlike IVT mRNAs, which function as a supplement to endogenous mRNAs, siRNAs play a role in cells as regulators and share some similarities with miRNAs. For example, both have similar physicochemical properties, and the mechanism of siRNA is the same as that of miRNA mimics. siRNA is derived from long dsRNA (without cervical-loop structures) in the cell *via* cleavage of long dsRNA by Dicer into siRNA. Subsequently, siRNA selectively targets and ultimately degrades complementary mRNA after being combined with RISC in the cytosol. However, unlike miRNAs, which show a different regulatory pattern, siRNAs function only to silence gene pathways. In addition, siRNA is fully complementary to mRNA 116, whereas miRNA is partially complementary to mRNA. Furthermore, siRNA is highly specific, and each type of siRNA targets only its complementary mRNA, while one type of miRNA can bind with multiple species of mRNA at the same time, and multiple mRNAs can target the same mRNA nucleotide sequence. This shows the specificity and high efficiency of siRNA in applications. siRNA and miRNA together are known as RNA interference (RNAi) 117, which leads to a highly efficient posttranscriptional gene silencing process in most eukaryotic cells. In RNAi therapy, siRNAs are synthesized *in vitro* and delivered into the cytoplasm, where they ultimately function as endogenous Dicer-cleaving products to degrade mRNA. In 2001, 21-nucleotide-long synthetic siRNAs were first successfully transfected into mammalian cells, resulting in successful gene silencing in transfected cells in studies by Tuschl and Gore 118; this provided new ideas for the treatment of numerous diseases such as ocular diseases, lung infections, skin diseases and cancer. Unmistakably, siRNA also plays an important role in bone regeneration, and its application will be discussed in detail in the following.

### siRNA in bone defect repair

In some studies, siRNA has been applied to silence a specific gene that inhibits the formation of osteocytes or facilitates the formation of osteoclasts with high specificity, which is a high efficiency method for bone defect repair. Earlier, scholars explored negative regulators of osteogenic differentiation to lay the foundation for later research. Kato et al 119 reported that silencing S100A4, a bone-related gene, could upregulate osteoblastic differentiation. Zhao and Ding 120 identified the main osteogenic suppressors in human mesenchymal stem cells through a high-throughput siRNA library screen, such as guanine nucleotide-binding protein (G protein) alpha subunit 1 (GNAS1). Cheema et al. 121 further proved that knocking out GNAS1 led to increased osteoblastic differentiation. Silencing the gene NOG with siRNA (siRNA-Noggin) enhanced the expression of BMP in cells 12. Tumor necrosis factor α (TNF-α) 122, casein kinase-2 interacting protein-1 (Ckip-1) 123, soluble VEGF receptor 1 (sFlt-1) 124, pleckstrin homology domain-containing family O member 1 (Plekho1) 125, WW domain-containing E3 ubiquitin protein ligase 1 (Wwp1) 126 and receptor activator of nuclear factor kappa B (RANK) 127 are negative regulators of bone formation and have been used as a target genes of siRNA silencing for bone regeneration.

### siRNA delivery to bone-related cells

Because siRNAs are vulnerable ribonucleotide strands, the delivery of naked siRNAs is less than ideal. Lipid-based transfection reagents, such as Lipofectamine 2000, are commonly used to deliver siRNA to cells. However, some highly differentiated cells, such as primary human osteoclasts, are hard to transfect, so more effective lipid carriers have been explored. Selinger 128 used FuGENE 6, an analogue of Lipofectamine 2000, for delivery to primary human osteoclasts; which was more efficient than using Lipofectamine 2000. In addition, Wang et al. 129 used biodegradable polyethylenimine (PEI-Et) to address the fact that PEI (PEI 25 kDa) lacks degradable linkages, which makes it too toxic for clinical applications. Due to the increase in bioindustry, various optimized siRNA-specific transfection reagents can be obtained commercially, including oligofectamine kits 119, the Xtreme-siRNA transfection reagent 120, 98N12(5) lipidoid 130, and the Ambion siPortTM Amine Transfection Agent 131. Therefore, for efficient delivery of siRNA to cells, the abovementioned reagents could be used. However, cell-free scaffolds need better optimized vectors to combinie siRNA in the complex environment of the body. Some synthetic copolymers are used in therapeutic devices with biocompatible and biodegradable properties to encapsulate siRNAs and release them in a controlled manner *in vitro* or *in vivo*. PLGA microparticles blended with PEI 127, 132, 133, PEG blended with PEI 12, 85, and poly-D-L-lactic acid (PLA) blended with PEI NPs 130 have been utilized to ameliorate the cytotoxicity of PEI. These carriers have been synthesized to deliver siRNA to cells on scaffolds, which is described in the vector part of **Table 3**.

### siRNA-based scaffolds in bone defect sites

In early studies of the delivery of siRNA to localized sites of interest *in vivo*, electroporation was used to deliver siRNA to cells 12, 134, which may result in electrical damage to cells. In contrast, chemical substances provide a milder means of delivery. Compared with mRNA and miRNA, siRNA applied in bone repair utilizes two very common delivery methods: systemic delivery for bone repair of osteoporosis and local scaffold-based delivery for bone defect repair. Bisphosphonate 135, (Asp)_8_ or (Asp)_6_ oligopeptide 136, 137, (AspSerSer)_6_ oligopeptide 138 and CH6 aptamer 125 have a high affinity for hydroxyapatite, which can be exploited for carrying siRNAs specifically to bone-formation surfaces to silence genes that facilitate osteoporotic phenotype. Functionalized molecules were combined with lipid NPs (LNPs), PLGA NPs or PEG NPs and then delivered to osteoporosis sites *via* intravenous injection for the recovery of bone loss in ovariectomized (OVX) rodent species. For bone defect repairing, local scaffold-based siRNA delivery has greater advantages because of its organ specificity, decreased drug dose accumulation, excellent bone tissue penetration, and cellular internalization in bone defect sites. siRNA scaffolds are summarized in **Table 3**. Hydrogel-, collagen- and CS-based scaffolds are popular choices for the delivery of siRNA as they are biodegradable, biocompatible, and able to entrap siRNA in the desired site for sufficient silencing time. The methods of delivering siRNA to bone defect sites with these scaffolds have been realized *via* cell-loaded scaffolds and cell-free scaffolds, which are the same with methods of delivery mRNA and miRNA, as shown in **Figure 3**. Interestingly, injectable CPC-augmented matrix scaffolds could continuously release siRNA over 50 days, as shown in a study by Wang et al. 132; however, data on the silencing efficiency over such a long release time are unavailable. Nguyen 139 showed the silencing effect of photocrosslinked dextran (DEX) hydrogel over 11 days. In view of the slowness of bone repair, the extension of the effective time of siRNAs is still worth exploring.

## Co-delivery of RNAs

The individual delivery of mRNA, miRNA, or siRNA has been described in numerous studies. In some cases, the delivery of multiple RNAs would achieve the suppression of undesirable proteins (*via* siRNA or miRNA) and the expression of desirable proteins (*via* mRNA or miRNA) 145 at the same time. The molecular weights and structures of miRNA (~25 kDa) and siRNA (~23 kDa) are similar 116, whereas mRNA is a 600-10,000-kDa nucleotide. Hence, a single biomaterial, containing both miRNA and siRNA is more suitable, although co-delivery with mRNA is more complex. Ball et al. 145 showed that the efficiency of using ionizable LNPs for co-delivering siRNA and mRNA could be improved by adjusting the proportions of the components (lipids, cholesterol, helper lipid and PEG) in the delivery vehicle. In addition, the ratio of the concentration of the siRNA and mRNA also affects the final internalization.

It is worth noting that the similar chemical properties of negatively charged nucleotides (DNAs or RNAs) simplifies the design of their delivery materials, but their co-delivery with proteins or drugs requires different designs according to the relevant physicochemical properties. For instance, PLGA NPs are positively charged on the surface after being modified with PEI, which could applied to the co-delivery of gene-gene, gene-protein or gene-drug combinations 146-149. The surfaces of PLGA NPs gained a positive charge after pretreatment with PEI. The modifying surface particles that were conjugated with SOX 9 pDNA and Cbfa-1 siRNA were subsequently co-transfected into hMSCs to enhance cartilage differentiation 147. As a result, both SOX 9 pDNA and Cbfa-1 siRNA were linked to the outer surface of the positively charged spheres through electrostatic interactions. In contrast, for co-delivery of RNAs and proteins to hMSCs, Cbfa-1 siRNAs were loaded onto the outer surfaces of PLGA NPs, whereas the SOX 9 protein was encapsulated into the particles 148. To induce osteogenic differentiation of hMSCs, Park et al. 150 used PLGA NPs encapsulating Runx2 protein and coated with BMP-2 pDNA. Besides PLGA NPs, cationic sterosomes 151, quantum dots (modified with β-cyclodextrin and Cys-Lys-Lys-Arg-Gly-Asp (CKKRGD) peptide) nanocarriers 152 and polymeric micelles 153 were exploited to co-deliver RNAs and drug molecules that promote bone healing (such as dexamethasone and simvastatin) *via* electrostatic or hydrophobic interactions to induce the osteogenic differentiation of MSCs.

## Advantages and disadvantages of using scaffolds for RNA delivery

The scaffold matrix provides not only a delivery platform for drugs, proteins or other reparative factors but also a structural support for infiltrating cells during bone regeneration. However, high dosing strategies require consideration of the fact that long-term production of therapeutic proteins is still needed at the desired sites. Efforts to repair injured bone *via* gene-enhanced scaffold matrixes began in the 1990s 154, and these matrixes could retain the expression of the gene for at least 6 weeks after implantation into osseous defects; this was applied for RNA-based bone regeneration. RNA-enhanced scaffolds, as reservoirs of RNAs, locally deliver RNA complexes to sites of interest to avoid unwanted release in other sites by using economical methods. Moreover, *via* degradation by hydrolytic or biological enzymes, scaffolds could be spatiotemporal release small molecules in a controlled manner 155. Encouragingly, numerous scaffolds have been designed to prolong the release time of RNAs 132, 139. Another important advantage of scaffolds used for RNA delivery is that scaffolds can protect RNA complexes from serum nucleases or other biological enzymes to prevent physiological degradation to provide long-term, high-efficiency therapy *in vivo* 60, 126.

However, the uncertainty of the scaffold degradation rate and the specific parameters of the RNAs released from the scaffolds affect the efficacy of the RNAs. Sometimes, the strong interactions between scaffolds and vectors can limit the release of RNAs. Therefore, the coimmobilization of vectors and cells in scaffolds to facilitate the effective cellular internalization of infiltrating cells *in situ* is challenging. In addition, weak electrostatic interactions or biodegradable linkages between RNA/vector complexes and scaffolds could be used to control the delivery of RNA/vector complexes. Although existing research findings show that methods for the controlled release of RNAs from scaffolds over an extended time period have been continuously improved *in vitro*, few studies have been able to achieve long-term controlled release of RNAs from scaffolds and effective function of RNAs in targeted cells *in vivo*, which are the major limitations of RNA-based therapy for clinical bone remodeling.

## Conclusions and perspectives

Recently, RNA-based treatment methods have evoked intense interest for application to the treatment of numerous diseases because of the specific advantages of RNA 156. RNA-based scaffolds for bone regeneration were discussed and summarized in this review, emphasizing the use of RNA to promote osteogenesis as therapeutic molecules loaded onto/into scaffolds. Among various biomaterials designed for the protection and delivery of RNAs, LNPs and polymer NPs are the main vectors used for carrying RNAs, and hydrogel and collagen are the most common scaffold materials used for the delivery of RNA complexes to bone sites *in vivo*. However, the use of RNA-based therapy in bone regeneration is still in its infancy and should be further addressed to meet clinical needs. First, bone repair is a long and complex process. Various injuries or different degrees of damage could lead to different healing effects in the clinic 157, which would present additional challenges for the successful utilization of RNA-based therapy. In addition, the effect the physical environment (mechanical properties, degree of roughness, concentrations of components, and the macro-shapes of scaffolds) and chemical factors (e.g., environmental pH) are important for bone remodeling processes during scaffold-based therapy for bone repair. However, these specific parameters were not explored during recent studies. For example, our previous study proved that difference in stiffness affect the osteogenic differentiation of MSCs in a 3D culture environment 158. How do differences in stiffness affect delivery of RNAs? If such factors play a role in the release and transfection of RNAs, they would facilitate the development of RNA-loaded scaffolds for superior treatment. Second, it has been shown that RNAs could be stably released for more than 50 days *in vitro* 132. The RNA delivery system tested by single cell line was available *in vitro*, but was not easy *in vivo*. Moreover, the long-term sustained release of RNAs is not equivalent to the long-term transfection of RNAs. Therefore, the extension of the effective time *in vivo* is still worth exploring. Finally, although many RNA delivery scaffolds have been explored for curing bone injury, a low-cost, wide-ranging and effective preparation method for RNA-based scaffolds has not yet been established. To facilitate the development of commercialized scaffolds, 3D printing technology might be introduced to fabricate RNA-based scaffolds. Moreover, some nanocarriers, e.g., AuNPs 159, may be considered for the loading of RNAs, which could draw lessons from the use of biomaterials in cancer drug delivery for successful bone defect healing.

## Figures and Tables

**Table 1:** List of mRNA-based scaffolds for bone healing

Scaffolds		Gene	Vector	Implantation	Time	Ref
**Hydrogel**	CPC/PLGA microsphere	EPO-cmRNA	Lipoplexes	*In vitro*	4 days	61
	Fibrin gel scaffold	BMP-2-cmRNA	Micro-macro biphasic calcium phosphate (MBCP) ceramic granules	*In vitro*	3 weeks	62
	Fibrin gel scaffold	hBMP-2-cmRNA	Lipoplexes	Non-critical femoral bone defect model in rats	2 weeks	27
**Collagen**	Collagen scaffold	BMP-2-cmRNA	Polyethylenimine (PEI)	Calvarial defects in rats	4 weeks	7
	Collagen sponge scaffold	BMP-2-cmRNA	Lipoplexes	Critical-sized mid-femoral defect in rats	8 weeks	28
	Collagen scaffold	BMP-2-cmRNA or BMP-9-cmRNA	PEI	Critical-sized defects in rats	4 weeks	37
	Collagen sponge scaffold	hBMP-2-cmRNA	Lipofectamine 2000	Non-critical femoral bone defect model in rats	2 weeks	60
**Other**	Sheep and porcine tissue	Luciferase-cmRNA	Lipoplexes/mag-lipoplexes	*In vitro*	24 hours	63

**Abbreviations**: PLGA: poly (lactic-co-glycolic acid)

**Table 2:** List of miRNA-based scaffolds for bone healing

Scaffolds		Gene	Vector	Implantation	Time	Ref
**Hydrogel**	hMSCs loaded PEG hydrogel	miRNA-20a	PEI	*In vitro*	4 weeks	86
	MAO Ti surface	miR-21	CS/hyaluronic acid NPs	*In vitro*	4 weeks	109
	Gel culture plate	miR-21	CS/hyaluronic acid NPs	*In vitro*	4 weeks	110
	CS/TPP/ hyaluronic acid NPs	Anti-miR-138	CS/TPP/hyaluronic acid NPs	*In vitro*	3 weeks	111
	Injection biomedical fbrin glue hydrogel mixed with BMSCs (post-transfection)	miR-5106	BGNCs	Critical-sized calvarial defect in rats	4 weeks	97
	PLLA scaffold	miR-26a	HP vector-PLGA microsphere	Critical-sized calvarial bone in mouse	8 weeks	98
	GP hydrogel scaffold	Anti-miR-138	CS NPs	Critical-sized calvarial defect in rats	8 weeks	99
	miR-335-5p-transfected BMSCs loaded-silk sacffold	miR-335-5p	Lipidoid nanoparticles	Critical-sized calvarial bone in mouse	5 weeks	112
**Collagen**	BMSCs (post-transfection) loaded collagen-based hydrogel	miR-34a	Lipofectamine 2000	The tibial defect model in rats	8 weeks	10
	Atelocollagen scaffold	Anti-miR-222	Lipofectamine	The refractory fracture model in rats	8 weeks	72
	BMSCs (post-transfection) loaded collagen scaffold	miR-148b	Silver NPs	Critical-sized calvarial defect in rats	8 weeks	93
**HA**	Transfected hMSCs loaded on HA/TCP ceramic powder	Anti-miR-138	Lipofectamine 2000	s.c. in mouse for ectopic bone formation	8 weeks	9
	Transfected hMSCs loaded HA/TCP scaffold	Anti-miR-34a	Lipofectamine 2000	s.c. in mouse for ectopic bone formation	8 weeks	71
	ASCs (post-transfection) loaded porous HA scaffold	miR-26a	Lipofectamine 2000	The tibial defect model in rats	12 weeks	104
	Transfected hMSCs loaded on HA/TCP scaffold	Anti-miR-138, anti-miR-432 or anti-miR-222	Lipofectamine 2000	s.c. in mouse for ectopic bone formation	8 weeks	105
	Collagen-nHA scaffold	Dy547-labeled miRNA	nHA	*In vitro*	1 week	106
	Collagen-nHA scaffold	Anti-miR-133a	nHA	*In vitro*	4 weeks	107
	Collagen-nHA scaffold	miR-16	nHA	*In vitro*	4 weeks	108
	CS/nHA/nano-zirconium dioxide scaffold	miR-590-5p	The X-treme gene transfection reagent	*In vitro*	1 week	113
**Others**	PCL scaffold	miR-148b	Silver NPs	Critical-sized calvarial bone in mouse	12 weeks	91
	PCL scaffold	Anti-miR-432 or anti-miR-222	Lipofectamine 2000	*In vitro*	2 weeks	105
	BMMSCs sheet	miR-122	Micro/macro titanium oxide	*In vitro*	8 weeks	114

**Abbreviations**: BGNCs: bioactive glass nanoclusters; CS: chitosan; GP: β-sodium glycerol phosphate; HA/TCP: hydroxyapatite/tricalcium phosphate; HP: hyperbranched polymer; MAO: microarc oxidation; nHA: coll-nano hydroxyapatite; PCL: polycaprolactone; PEG: polyethylene glycol; PLLA: poly-l-lactide; TPP: tripolyphosphate.

**Table 3:** List of siRNA-based scaffolds for bone healing

Scaffolds		Gene	Vector	Implantation	Time	Ref
Hydrogel	PEG hydrogel scaffolds	siRNA	PEI	*In vitro*	4 weeks	87
	Injectable CPC augmentation matrix scaffolds	siRNA-RANK	PLGA microparticles	*In vitro*	12 days	132
	DEX hydrogel scaffolds	siRNA-GFP	PEI	*In vitro*	17 days	139
	Dual-crosslinked photodegradable hydrogels scaffolds	siRNA-Noggin	PEI	*In vitro*	2 weeks	140
	Fibrin hydrogel scaffolds	siRNA-Noggin	Lipofectamine 2000	*In vitro*	48 hours	141
	PEG hydrogel scaffolds	siRNA-Noggin	PEI	Calvarial bone defect model in rats	12 weeks	13
	SFCS scaffolds	siRNA-GNAS1 siRNA-PHD2	siPort amine transfection agent	Subcutaneous transplantation for ectopic bone formation	10 weeks	130
	PLA-DX-PEG pellet	siRNA-Noggin	PLA-DX-PEG polymer	The left dorsal muscle pouches of mouse for ectopic bone formation	7 days	142
	PLGA-PEG-PLA-DM hydrogel scaffolds	siRNA-Cy5	Polymer diblock NPs	Femur fracture model	4 weeks	143
	PLLA scaffolds	siRNA-Sema4d	Asp8-Stearyl-R8	Femoral defect model in OVX rats	8 weeks	144
Collagen	Collagen sponge disk scaffolds	siRNA-Noggin	Lipofectamine 2000 (*in vitro*) Electroporation (*in vivo*)	Dorsal muscular tissue in the Male ICR mice for ectopic bone induction	2 weeks	12
CS	CS sponge scaffolds	siRNA-Ckip-1 and siRNA-Flt-1	Lipofectamine™ 2000	Calvarial bone defect model in rats	12 weeks	124

**Abbreviations:** Ckip-1: casein kinase 2 interaction protein 1; CPC: calcium phosphatecement; DEX: dextran; Flt-1: soluble VEGF receptor 1; GFP: green fluorescent protein; GNAS1: guanine nucleotide-binding protein alpha subunit 1; PEG-PLA-DM : poly(ethylene glycol)-poly(lactic acid)-dimethacrylate; PEI: polyetherimide; PHD2: prolyl hydroxylase domaincontaining protein 2; PLA-DX-PEG: poly-D,L-lactic acid-p-dioxanonepolyethylene glycol block co-polymer; PLLA: poly-L-lactic acid; SFCS: silk fibroin-chitosan.

## Abbreviations

3D: three-dimensional
ALP: alkaline phosphatase
AMSCs: adipose mesenchymal stem cells
BGNCs: bioactive glass nanoclusters
BMP-2: bone morphogenetic protein-2
BMSCs: bone marrow mesenchymal stem cells
ceRNAs: competing endogenous RNAs
Ckip-1: casein kinase-2 interacting protein-1
cmRNA: chemically modified messenger RNA
CPC: calcium phosphate cement
CS: chitosan
DEX: dextran
eGFP: enhanced-green fluorescent protein
GNAS1: guanine nucleotide-binding protein alpha subunit 1
GP: β-sodium glycerol phosphate
HA: hydroxyapatite
hBMP-2: human BMP-2
hMSCs: human mesenchymal stem cells
IVT mRNA: messenger RNA-synthesized *in vitro* transcription
lncRNA: long non-coding RNA
LNPs: lipid-nanoparticles
MBCP: micro-macro biphasic calcium phosphate
miRNA: micro RNA
mRNA: messenger RNA
MSCs: mesenchymal stem cells
NPs: nanoparticles
ORF: open reading frame
OVX: ovariectomized
pDNA: plasmid DNA
PEI: polyethyleneimine
Plekho1: leckstrin homology domain-containing family O member 1
PLGA: poly (lactic-co-glycolic acid)
PLL: poly-L-lysine
pre-miRNA: precursor miRNA
pri-miRNA: primary miRNA
RANK: receptor activator of nuclear factor kappa B
RAS: activity and alizarin red staining
RISC: RNA-induced silencing complex
siRNA: short interfering RNA
TCP: tricalcium phosphate
TISU: short untranslated regions
TNF-α: tumor necrosis factor alpha
tRNA: transfer RNA
UTR: untranslated region

## References

[1] Roseti L, Parisi V, Petretta M, Cavallo C, Desando G, Bartolotti I (2017). Scaffolds for bone tissue engineering: state of the art and new perspectives. Mater Sci Eng C Mater Biol Appl.

[2] Deng C, Zhu H, Li J, Feng C, Yao Q, Wang L (2018). Bioactive scaffolds for regeneration of cartilage and subchondral bone interface. Theranostics.

[3] Tang Q, Hu Z, Jin H, Zheng G, Yu X, Wu G (2019). Microporous polysaccharide multilayer coated BCP composite scaffolds with immobilised calcitriol promote osteoporotic bone regeneration both in vitro and in vivo. Theranostics.

[4] Fernandez de Grado G, Keller L, Idoux-Gillet Y, Wagner Q, Musset AM, Benkirane-Jessel N (2018). Bone substitutes: a review of their characteristics, clinical use, and perspectives for large bone defects management. J Tissue Eng.

[5] D'Mello S, Atluri K, Geary SM, Hong L, Elangovan S, Salem AK (2017). Bone regeneration using gene-activated matrices. AAPS J.

[6] Berendsen AD, Olsen BR (2015). Bone development. Bone.

[7] Elangovan S, Khorsand B, Do AV, Hong L, Dewerth A, Kormann M (2015). Chemically modified RNA activated matrices enhance bone regeneration. J Control Release.

[8] Lin CY, Crowley ST, Uchida S, Komaki Y, Kataoka K, Itaka K (2019). Treatment of intervertebral disk disease by the administration of mRNA encoding a cartilage-anabolic transcription factor. Mol Ther Nucleic Acids.

[9] Eskildsen T, Taipaleenmäki H, Stenvang J, Abdallah BM, Ditzel N, Nossent AY (2011). MicroRNA-138 regulates osteogenic differentiation of human stromal (mesenchymal) stem cells in vivo. Proc Natl Acad Sci U S A.

[10] Liu H, Dong Y, Feng X, Li L, Jiao Y, Bai S (2019). miR-34a promotes bone regeneration in irradiated bone defects by enhancing osteoblastic differentiation of mesenchymal stromal cells in rats. Stem Cell Res Ther.

[11] Hao F, Lee RJ, Zhong L, Dong S, Yang C, Teng L (2019). Hybrid micelles containing methotrexate-conjugated polymer and co-loaded with microRNA-124 for rheumatoid arthritis therapy. Theranostics.

[12] Takayama K, Suzuki A, Manaka T, Taguchi S, Hashimoto Y, Imai Y (2009). RNA interference for noggin enhances the biological activity of bone morphogenetic proteins in vivo and in vitro. J Bone Miner Metab.

[13] Nguyen MK, Jeon O, Dang PN, Huynh CT, Varghai D, Riazi H (2018). RNA interfering molecule delivery from in situ forming biodegradable hydrogels for enhancement of bone formation in rat calvarial bone defects. Acta Biomater.

[14] Zhu L, Xu PC (2013). Downregulated lncRNA-ANCR promotes osteoblast differentiation by targeting EZH2 and regulating Runx2 expression. Biochem Biophys Res Commun.

[15] Hu K, Jiang W, Sun H, Li Z, Rong G, Yin Z (2019). Long noncoding RNA ZBED3-AS1 induces the differentiation of mesenchymal stem cells and enhances bone regeneration by repressing IL-1β via Wnt/β-catenin signaling pathway. J Cell Physiol.

[16] Zhang W, Chen L, Wu J, Li J, Zhang X, Xiang Y (2019). Long noncoding RNA TUG1 inhibits osteogenesis of bone marrow mesenchymal stem cells via Smad5 after irradiation. Theranostics.

[17] Zhang J, Hao X, Yin M, Xu T, Guo F (2019). Long non-coding RNA in osteogenesis: a new world to be explored. Bone Joint Res.

[18] Diomede F, Merciaro I, Martinotti S, Cavalcanti MF, Caputi S, Mazzon E (2016). miR-2861 is involved in osteogenic commitment of human periodontal ligament stem cells grown onto 3D scaffold. J Biol Regul Homeost Agents.

[19] Liu X (2016). Bone site-specific delivery of siRNA. J Biomed Res.

[20] Kwon H, Kim M, Seo Y, Moon YS, Lee HJ, Lee K (2018). Emergence of synthetic mRNA: in vitro synthesis of mRNA and its applications in regenerative medicine. Biomaterials.

[21] Arriaga MA, Ding MH, Gutierrez AS, Chew SA (2019). The application of microRNAs in biomaterial scaffold-based therapies for bone tissue engineering. Biotechnol J.

[22] Zhao W, Hou X, Vick OG, Dong Y (2019). RNA delivery biomaterials for the treatment of genetic and rare diseases. Biomaterials.

[23] Yamamoto A, Kormann M, Rosenecker J, Rudolph C (2009). Current prospects for mRNA gene delivery. Eur J Pharm Biopharm.

[24] Zou S, Scarfo K, Nantz MH, Hecker JG (2010). Lipid-mediated delivery of RNA is more efficient than delivery of DNA in non-dividing cells. Int J Pharm.

[25] Badieyan ZS, Evans T (2019). Concise review: application of chemically modified mRNA in ell fate conversion and tissue engineering. Stem Cells Transl Med.

[26] Tavernier G, Andries O, Demeester J, Sanders NN, De Smedt SC, Rejman J (2011). mRNA as gene therapeutic: how to control protein expression. J Control Release.

[27] Balmayor ER, Geiger JP, Aneja MK, Berezhanskyy T, Utzinger M, Mykhaylyk O (2016). Chemically modified RNA induces osteogenesis of stem cells and human tissue explants as well as accelerates bone healing in rats. Biomaterials.

[28] Zhang W, De La Vega RE, Coenen MJ, Müller SA, Peniche Silva CJ (2019). An improved, chemically modified RNA encoding BMP-2 enhances osteogenesis in vitro and in vivo. Tissue Eng Part A.

[29] Lockard RE, Lingrel JB (1969). The synthesis of mousehemoglobin beta-chains in a rabbit reticulocyte cellfree system programmed with mouse reticulocyte 9S RNA. Biochem Biophys Res Commun.

[30] Hajj KA, Whitehead KA (2017). Tools for translation: non-viral materials for therapeutic mRNA delivery. Nature.

[31] Van Tendeloo VF, Ponsaerts P, Lardon F, Nijs G, Lenjou M, Van Broeckhoven C (2001). Highly efficient gene delivery by mRNA electroporation in human hematopoietic cells: superiority to lipofection and passive pulsing of mRNA and to electroporation of plasmid cDNA for tumor antigen loading of dendritic cells. Blood.

[32] Qiu P, Ziegelhoffer P, Sun J, Yang NS (1996). Gene gun delivery of mRNA in situ results in efficient transgene expression and genetic immunization. Gene Ther.

[33] Layden MJ, Röttinger E, Wolenski FS, Gilmore TD, Martindale MQ (2013). Microinjection of mRNA or morpholinos for reverse genetic analysis in the starlet sea anemone, Nematostella vectensis. Nat Protoc.

[34] Probst J, Weide B, Scheel B, Pichler BJ, Hoerr I, Rammensee HG (2007). Spontaneous cellular uptake of exogenous messenger RNA in vivo is nucleic acid-specific, saturable and ion dependent. Gene Ther.

[35] Yu Z, Qian J, Wu J, Gao J, Zhang M (2012). Allogeneic mRNA-based electrotransfection of autologous dendritic cells and specific antitumor effects against osteosarcoma in rats. Med Oncol.

[36] Kauffman KJ, Webber MJ, Anderson DG (2016). Materials for non-viral intracellular delivery of messenger RNA therapeutics. J Control Release.

[37] Khorsand B, Elangovan S, Hong L, Dewerth A, Kormann MS, Salem AK (2017). A comparative study of the bone regenerative effect of chemically modified RNA encoding BMP-2 or BMP-9. AAPS J.

[38] Farrell LL, Pepin J, Kucharski C, Lin X, Xu Z, Uludag H (2007). A comparison of the effectiveness of cationic polymers poly-L-lysine (PLL) and polyethylenimine (PEI) for non-viral delivery of plasmid DNA to bone marrow stromal cells (BMSC). Eur J Pharm Biopharm.

[39] Kaczmarek JC, Patel AK, Kauffman KJ, Fenton OS, Webber MJ, Heartlein MW (2016). Polymer-lipid nanoparticles for systemic delivery of mRNA to the lungs. Angew Chem Int Ed Engl.

[40] Fan YN, Li M, Luo YL, Chen Q, Wang L, Zhang HB (2018). Cationic lipid-assisted nanoparticles for delivery of mRNA cancer vaccine. Biomater Sci.

[41] Patel S, Ryals RC, Weller KK, Pennesi ME, Sahay G (2019). Lipid nanoparticles for delivery of messenger RNA to the back of the eye. J Control Release.

[42] Liu J, Chang J, Jiang Y, Meng X, Sun T, Mao L (2019). Fast and efficient CRISPR/Cas9 genome editing in vivo enabled by bioreducible lipid and messenger RNA nanoparticles. Adv Mater.

[43] Guan S, Rosenecker J (2017). Nanotechnologies in delivery of mRNA therapeutics using nonviral vector-based delivery systems. Gene Ther.

[44] Petsch B, Schnee M, Vogel AB, Lange E, Hoffmann B, Voss D (2012). Protective efficacy of in vitro synthesized, specific mRNA vaccines against influenza a virus infection. Nat Biotechnol.

[45] Benencia F (2014). Antigen-specific mRNA transfection of autologous dendritic cells. Methods Mol Biol.

[46] Bahl K, Senn JJ, Yuzhakov O, Bulychev A, Brito LA, Hassett KJ (2017). Preclinical and clinical demonstration of immunogenicity by mRNA vaccines against H10N8 and H7N9 influenza viruses. Mol Ther.

[47] Pardi N, Secreto AJ, Shan X, Debonera F, Glover J, Yi Y (2017). Administration of nucleoside-modified mRNA encoding broadly neutralizing antibody protects humanized mice from HIV-1 challenge. Nat Commun.

[48] Le Moignic A, Malard V, Benvegnu T, Lemiègre L, Berchel M, Jaffrès PA (2018). Preclinical evaluation of mRNA trimannosylated lipopolyplexes as therapeutic cancer vaccines targeting dendritic cells. J Control Release.

[49] Abraham MK, Nolte A, Reus R, Behring A, Zengerle D, Avci-Adali M (2015). In vitro study of a novel stent coating using modified CD39 messenger RNA to potentially reduce stent angioplasty-associated complications. PLoS One.

[50] Tanaka H, Nakatani T, Furihata T, Tange K, Nakai Y, Yoshioka H (2018). In vivo introduction of mRNA encapsulated in lipid nanoparticles to brain neuronal cells and astrocytes via intracerebroventricular administration. Mol Pharm.

[51] Golombek S, Pilz M, Steinle H, Kochba E, Levin Y, Lunter D (2018). Intradermal delivery of synthetic mRNA using hollow microneedles for efficient and rapid production of exogenous proteins in skin. Mol Ther Nucleic Acids.

[52] Huang CL, Leblond AL, Turner EC, Kumar AH, Martin K, Whelan D (2015). Synthetic chemically modified mRNA-based delivery of cytoprotective factor promotes early cardiomyocyte survival post-acute myocardial infarction. Mol Pharm.

[53] Awasthi S, Hook LM, Pardi N, Wang F, Myles A, Cancro MP (2019). Nucleoside-modified mRNA encoding HSV-2 glycoproteins C, D, and E prevents clinical and subclinical genital herpes.

[54] Park SY, Kim KH, Kim S, Lee YM, Seol YJ (2019). BMP-2 gene delivery-based bone regeneration in dentistry. Pharmaceutics.

[55] Krötz F, de Wit C, Sohn HY, Zahler S, Gloe T, Pohl U (2003). Magnetofection a highly efficient tool for antisense oligonucleotide delivery in vitro and in vivo. Mol Ther.

[56] Mykhaylyk O1, Zelphati O, Rosenecker J, Plank C (2008). siRNA delivery by magnetofection. Curr Opin Mol Ther.

[57] Vosen S, Rieck S, Heidsieck A, Mykhaylyk O, Zimmermann K, Plank C (2016). Improvement of vascular function by magnetic nanoparticle-assisted circumferential gene transfer into the native endothelium. J Control Release.

[58] Raisin S, Belamie E, Morille M (2016). Non-viral gene activated matrices for mesenchymal stem cells based tissue engineering of bone and cartilage. Biomaterials.

[59] Glowacki J, Mizuno S (2008). Collagen scaffolds for tissue engineering. Biopolymers.

[60] Badieyan ZS, Berezhanskyy T, Utzinger M, Aneja MK, Emrich D, Erben R (2016). Transcript-activated collagen matrix as sustained mRNA delivery system for bone regeneration. J Control Release.

[61] Utzinger M, Jarzebinska A, Haag N, Schweizer M, Winter G, Dohmen C (2017). cmRNA/lipoplex encapsulation in PLGA microspheres enables transfection via calcium phosphate cement (CPC)/PLGA composites. J Control Release.

[62] Balmayor ER, Geiger JP, Koch C, Aneja MK, van Griensven M, Rudolph C (2017). Modified mRNA for BMP-2 in combination with biomaterials serves as a transcript-activated matrix for effectively inducing osteogenic pathways in stem cell. Stem Cells Dev.

[63] Badieyan ZS, Pasewald T, Mykhaylyk O, Rudolph C, Plank C (2017). Efficient ex vivo delivery of chemically modified messenger RNA using lipofection and magnetofection. Biochem Biophys Res Commun.

[64] Fang S, Deng Y, Gu P, Fan X (2015). MicroRNAs regulate bone development and regeneration. Int J Mol Sci.

[65] Nakasa T, Yoshizuka M, Andry Usman M, Elbadry Mahmoud E, Ochi M (2015). MicroRNAs and bone regeneration. Current Genomics.

[66] Li J, Tan S, Kooger R, Zhang C, Zhang Y (2014). MicroRNAs as novel biological targets for detection and regulation. Chem Soc Rev.

[67] Taipaleenmäki H, Bjerre Hokland L, Chen L, Kauppinen S, Kassem M (2012). Micro-RNAs: targets for enhancing osteoblast differentiation and bone formation. Eur J Endocrinol.

[68] Curtin CM, Castaño IM1, O'Brien FJ (2018). Scaffold-based microRNA therapies in regenerative medicine and cancer. Adv Healthc Mater.

[69] Lim LP, Lau NC, Garrett-Engele P, Grimson A, Schelter JM, Castle J (2005). Microarray analysis shows that some microRNAs downregulate large numbers of target mRNAs. Nature.

[70] Pu M, Chen J, Tao Z, Miao L, Qi X, Wang Y (2019). Regulatory network of miRNA on its target: coordination between transcriptional and post-transcriptional regulation of gene expression. Cell Mol Life Sci.

[71] Chen L, Holmstrøm K, Qiu W, Ditzel N, Shi K, Hokland L (2014). MicroRNA-34a inhibits osteoblast differentiation and in vivo bone formation of human stromal stem cells. Stem Cells.

[72] Yoshizuka M, Nakasa T, Kawanishi Y, Hachisuka S, Furuta T, Miyaki S (2016). Inhibition of microRNA-222 expression accelerates bone healing with enhancement of osteogenesis, chondrogenesis, and angiogenesis in a rat refractory fracture model. J Orthop Sci.

[73] Kang S, Im K, Baek J, Yoon S, Min H (2014). Macro and small over micro: macromolecules and small molecules that regulate microRNAs. Chembiochem.

[74] Phimphilai M, Zhao Z, Boules H, Roca H, Franceschi RT (2006). BMP signaling is required for RUNX2-dependent induction of the osteoblast phenotype. J Bone Miner Res.

[75] Liao YH, Chang YH, Sung LY, Li KC, Yeh CL, Yen TC (2014). Osteogenic differentiation of adipose-derived stem cells and calvarial defect repair using baculovirus-mediated co-expression of BMP-2 and miR-148b. Biomaterials.

[76] Li KC, Lo SC, Sung LY, Liao YH, Chang YH, Hu YC (2017). Improved calvarial bone repair by hASCs engineered with Cre/loxP-based baculovirus conferring prolonged BMP-2 and MiR-148b co-expression. J Tissue Eng Regen Med.

[77] Liu L, Shu S, Cheung GS, Wei X (2016). Effect of miR-146a/bFGF/PEG-PEI nanoparticles on inflammation response and tissue regeneration of human dental pulp cells. Biomed Res Int.

[78] Xie Q, Wei W, Ruan J, Ding Y, Zhuang A, Bi X (2017). Effects of miR-146a on the osteogenesis of adipose-derived mesenchymal stem cells and bone regeneration. Sci Rep.

[79] Kureel J, Dixit M, Tyagi AM, Mansoori MN, Srivastava K, Raghuvanshi A (2014). miR-542-3p suppresses osteoblast cell proliferation and differentiation, targets BMP-7 signaling and inhibits bone formation. Cell Death Dis.

[80] Huang S, Wang S, Bian C, Yang Z, Zhou H, Zeng Y (2012). Upregulation of miR-22 promotes osteogenic differentiation and inhibits adipogenic differentiation of uman adipose tissue-derived mesenchymal stem cells by repressing HDAC6 protein expression. Stem Cells Dev.

[81] Zuo B, Zhu J, Li J, Wang C, Zhao X, Cai G (2015). microRNA-103a functions as a mechanosensitive microRNA to inhibit bone formation through targeting Runx2. J Bone Miner Res.

[82] Kureel J, John AA, Dixit M, Singh D (2017). MicroRNA-467g inhibits new bone regeneration by targeting Ihh/Runx-2 signaling. Int J Biochem Cell Biol.

[83] Xie Q, Wang Z, Zhou H, Yu Z, Huang Y, Sun H (2016). The role of miR-135-modified adipose-derived mesenchymal stem cells in bone regeneration. Biomaterials.

[84] Luzi E, Marini F, Sala SC, Tognarini I, Galli G, Brandi ML (2008). Osteogenic differentiation of human adipose tissue-derived stem cells is modulated by the miR-26a targeting of the SMAD1 transcription factor. J Bone Miner Res.

[85] Trompeter HI1, Dreesen J, Hermann E, Iwaniuk KM, Hafner M, Renwick N (2013). MicroRNAs miR-26a, miR-26b, and miR-29b accelerate osteogenic differentiation of unrestricted somatic stem cells from human cord blood. BMC Genomics.

[86] Nguyen MK, Jeon O, Krebs MD, Schapira D, Alsberg E (2014). Sustained localized presentation of RNA interfering molecules from in situ forming hydrogels to guide stem cell osteogenic differentiation. Biomaterials.

[87] Li Z, Hassan MQ, Volinia S, van Wijnen AJ, Stein JL, Croce CM (2008). A microRNA signature for a BMP2-induced osteoblast lineage commitment program. Proc Natl Acad Sci U S A.

[88] Mizuno Y, Yagi K, Tokuzawa Y, Kanesaki-Yatsuka Y, Suda T, Katagiri T (2008). miR-125b inhibits osteoblastic differentiation by down-regulation of cell proliferation. Biochem Biophys Res Commun.

[89] Hu R, Liu W, Li H, Yang L, Chen C, Xia ZY (2011). A Runx2/miR-3960/miR-2861 regulatory feedback loop during mouse osteoblast differentiation. J Biol Chem.

[90] Huang J, Zhao L, Xing L, Chen D (2010). MicroRNA-204 regulates Runx2 protein expression and mesenchymal progenitor cell differentiation. Stem Cells.

[91] Trompeter HI, Dreesen J, Hermann E, Iwaniuk KM, Hafner M, Renwick N (2013). MicroRNAs miR-26a, miR-26b, and miR-29b accelerate osteogenic differentiation of unrestricted somatic stem cells from human cord blood. BMC Genomics.

[92] Qureshi AT, Doyle A, Chen C, Coulon D, Dasa V, Del Piero F (2015). Photoactivated miR-148b-nanoparticle conjugates improve closure of critical size mouse calvarial defects. Acta Biomater.

[93] Moncal KK, Aydin RST, Abu-Laban M, Heo DN, Rizk E, Tucker SM (2019). Collagen-infilled 3D printed scaffolds loaded with miR-148b-transfected bone marrow stem cells improve calvarial bone regeneration in rats. Mater Sci Eng C Mater Biol Appl.

[94] Gibbs DM, Black CR, Dawson JI, Oreffo RO (2016). A review of hydrogel use in fracture healing and bone regeneration. J Tissue Eng Regen Med.

[95] Saravanan S, Vimalraj S, Thanikaivelan P, Banudevi S, Manivasagam G (2019). A review on injectable chitosan/beta glycerophosphate hydrogels for bone tissue regeneration. Int J Biol Macromol.

[96] Li Y, Fan L, Liu S, Liu W, Zhang H, Zhou T (2013). The promotion of bone regeneration through positive regulation of angiogenic-osteogenic coupling using microRNA-26a. Biomaterials.

[97] Xue Y, Guo Y, Yu M, Wang M, Ma PX, Lei B (2017). Monodispersed bioactive glass nanoclusters with ultralarge pores and intrinsic exceptionally high miRNA loading for effciently enhancing bone regeneration.

[98] Zhang X, Li Y, Chen YE, Chen J, Ma PX (2016). Cell-free 3D scaffold with two-stage delivery of miRNA-26a to regenerate critical-sized bone defects. Nat Commun.

[99] Wu G, Feng C, Quan J, Wang Z, Wei W (2018). In situ controlled release of stromal cell-derived factor-1α and antimiR-138 for on-demand cranial bone regeneration. Carbohydr Polym.

[100] Žižka R, Šedý J (2017). Paradigm shift from stem cells to cell-free regenerative endodontic procedures: a critical review. Stem Cells Dev.

[101] Ma H, Feng C, Chang J, Wu C (2018). 3D-printed bioceramic scaffolds: From bone tissue engineering to tumor therapy. Acta Biomater.

[102] Aldaadaa A, Owji N, Knowles J (2018). Three-dimensional printing in maxillofacial surgery: hype versus reality. J Tissue Eng.

[103] Szcześ A, Hołysz L, Chibowski E (2017). Synthesis of hydroxyapatite for biomedical applications. Adv Colloid Interface Sci.

[104] Wang Z, Zhang D, Hu Z, Cheng J, Zhuo C, Fang X (2015). microRNA-26a-modified adipose-derived stem cells incorporated with a porous hydroxyapatite scaffold improve the repair of bone defects. Mol Med Rep.

[105] Chang CC, Venø MT, Chen L, Ditzel N, Le DQS, Dillschneider P (2018). Global microRNA profiling in human bone marrow skeletal-stromal or mesenchymal-stem cells identified candidates for bone regeneration. Mol Ther.

[106] Mencía Castaño I, Curtin CM, Shaw G, Murphy JM, Duffy GP, O'Brien FJ (2015). A novel collagen-nanohydroxyapatite microRNA-activated scaffold for tissue engineering applications capable of efficient delivery of both miR-mimics and antagomiRs to human mesenchymal stem cells. J Control Release.

[107] Mencía Castaño I, Curtin CM, Duffy GP, O'Brien FJ (2016). Next generation bone tissue engineering: non-viral miR-133a inhibition using collagen-nanohydroxyapatite scaffolds rapidly enhances osteogenesis. Sci Rep.

[108] Mencía Castaño I, Curtin CM, Duffy GP, O'Brien FJ (2019). Harnessing a novel inhibitory role of miR-16 in osteogenesis by human mesenchymal stem cells for advanced scaffold-based bone tissue engineering. Tissue Eng Part A.

[109] Wang Z, Wu G, Feng Z, Bai S, Dong Y, Wu G (2015). Microarc-oxidized titanium surfaces functionalized with microrNa-21-loaded chitosan/hyaluronic acid nanoparticles promote the osteogenic differentiation of human bone marrow mesenchymal stem cells. Int J Nanomedicine.

[110] Wang Z, Wu G, Wei M, Liu Q, Zhou J, Qin T (2016). Improving the osteogenesis of human bone marrow mesenchymal stem cell sheets by microrNa-21- loaded chitosan/hyaluronic acid nanoparticles via reverse transfection. Int J Nanomedicine.

[111] Wu G, Feng C, Hui G, Wang Z, Tan J, Luo L (2016). Improving the osteogenesis of rat mesenchymal stem cells by chitosan-based-microRNA nanoparticles. Carbohydr Polym.

[112] Sui L, Wang M, Han Q, Yu L, Zhang L, Zheng L (2018). A novel Lipidoid-MicroRNA formulation promotes calvarial bone regeneration. Biomaterials.

[113] Balagangadharan K, Viji Chandran S, Arumugam B, Saravanan S, Devanand Venkatasubbu G, Selvamurugan N (2018). Chitosan/nano-hydroxyapatite/nano-zirconium dioxide scaffolds with miR-590-5p for bone regeneration. Int J Biol Macromol.

[114] Shao D, Wang C, Sun Y, Cui L (2018). Effects of oral implants with miR-122-modified cell sheets on rat bone marrow mesenchymal stem cells. Mol Med Rep.

[115] Wang J, Lu Z, Wientjes MG, Au JL (2010). Delivery of siRNA therapeutics: barriers and carriers. AAPS J.

[116] Lam JK, Chow MY, Zhang Y, Leung SW (2015). siRNA versus miRNA as therapeutics for gene silencing. Mol Ther Nucleic Acids.

[117] De Fougerolles A, Vornlocher HP, Maraganore J, Lieberman J (2007). Interfering with disease: a progress report on siRNA-based therapeutics. Nat Rev Drug Discov.

[118] Nikam RR, Gore KR (2018). Journey of siRNA: clinical developments and targeted delivery. Nucleic Acid Ther.

[119] Kato C, Kojima T, Komaki M, Mimori K, Duarte WR, Takenaga K (2005). S100A4 inhibition by RNAi up-regulates osteoblast related genes in periodontal ligament cell. Biochem Biophys Res Commun.

[120] Zhao Y, Ding S (2007). A high-throughput siRNA library screen identifies osteogenic suppressors in human mesenchymal stem cells. Proc Natl Acad Sci U S A.

[121] Cheema SK, Chen E, Shea LD, Mathur AB (2007). Regulation and guidance of cell behavior for tissue regeneration via the siRNA mechanism. Wound Repair Regen.

[122] Guo HH, Yu CC, Sun SX, Ma XJ, Yang XC, Sun KN (2013). Adenovirus-mediated siRNA targeting TNF-α and overexpression of bone morphogenetic protein-2 promotes early osteoblast differentiation on a cell model of Ti particle-induced inflammatory response in vitro. Braz J Med Biol Res.

[123] Zhou ZC, Che L, Kong L, Lei DL, Liu R, Yang XJ (2017). CKIP-1 silencing promotes new bone formation in rat mandibular distraction osteogenesis. Oral Surg Oral Med Oral Pathol Oral Radiol.

[124] Jia S, Yang X, Song W, Wang L, Fang K, Hu Z (2014). Incorporation of osteogenic and angiogenic small interfering rNas into chitosan sponge for bone tissue engineering. Int J Nanomedicine.

[125] Liang C, Guo B, Wu H, Shao N, Li D, Liu J (2015). Aptamer-functionalized lipid nanoparticles targeting osteoblasts as a novel RNA interference-based bone anabolic strategy. Nat Med.

[126] Wang Y, Malcolm DW, Benoit DSW (2017). Controlled and sustained delivery of siRNA/NPs from hydrogels expedites bone fracture healing. Biomaterials.

[127] Sezlev Bilecen D, Uludag H, Hasirci V (2019). Development of PEI-RANK siRNA complex Loaded PLGA nanocapsules for the treatment of osteoporosis. Tissue Eng Part A.

[128] Selinger CI, Day CJ, Morrison NA (2005). Optimized transfection of Diced siRNA into mature primary human osteoclasts: inhibition of cathepsin K mediated bone resorption by siRNA. J Cell Biochem.

[129] Wang C, Yuan W, Xiao F, Gan Y, Zhao X, Zhai Z (2017). Biscarbamate cross-linked low-molecular-weight polyethylenimine for delivering anti-chordin siRNA into human mesenchymal stem cells for improving bone regeneration. Front Pharmacol.

[130] Zoldan J, Lytton-Jean AK, Karagiannis ED, Deiorio-Haggar K, Bellan LM, Langer R (2011). Directing human embryonic stem cell differentiation by non-viral delivery of siRNA in 3D culture. Biomaterials.

[131] Ríos CN, Skoracki RJ, Mathur AB (2012). GNAS1 and PHD2 short-interfering RNA support bone regeneration in vitro and in an in vivo sheep model. Clin Orthop Relat Res.

[132] Wang Y, Tran KK, Shen H, Grainger DW (2012). Selective local delivery of RANK siRNA to bone phagocytes using bone augmentation biomaterials. Biomaterials.

[133] Jeon SY, Park JS, Yang HN, Woo DG, Park KH (2012). Co-delivery of SOX9 genes and anti-Cbfa-1 siRNA coated onto PLGA nanoparticles for chondrogenesis of human MSCs. Biomaterials.

[134] Inoue A, Takahashi KA, Mazda O, Terauchi R, Arai Y, Kishida T (2005). Electro-transfer of small interfering RNA ameliorated arthritis in rats. Biochem Biophys Res Commun.

[135] Giger EV, Castagner B, Räikkönen J, Mönkkönen J, Leroux JC (2013). siRNA transfection with calcium phosphate nanoparticles stabilized with PEGylated chelators. Adv Healthc Mater.

[136] Wang D, Miller SC, Shlyakhtenko LS, Portillo AM, Liu XM, Papangkorn K (2007). Osteotropic peptide that differentiates functional domains of the skeleton. Bioconjug Chem.

[137] Zhang Y, Wei L, Miron RJ, Shi B, Bian Z (2015). Anabolic bone formation via a site-specific bone-targeting elivery system by interfering with semaphorin 4D expression. J Bone Miner Res.

[138] Zhang G, Guo B, Wu H, Tang T, Zhang BT, Zheng L (2012). A delivery system targeting bone formation surfaces to facilitate RNAi-based anabolic therapy. Nat Med.

[139] Nguyen K, Dang PN, Alsberg E (2013). Functionalized, biodegradable hydrogels for control over sustained and localized siRNA delivery to incorporated and surrounding cells. Acta Biomater.

[140] Huynh CT, Nguyen MK, Naris M, Tonga GY, Rotello VM, Alsberg E (2016). Light-triggered RNA release and induction of hMSC osteogenesis via photodegradable, dual-crosslinked hydrogels. Nanomedicine (Lond).

[141] Kowalczewski CJ, Saul JM (2015). Surface-mediated delivery of siRNA from fibrin hydrogels for knockdown of the BMP-2 binding antagonist noggin. Acta Biomater.

[142] Manaka T, Suzuki A, Takayama K, Imai Y, Nakamura H, Takaoka K (2011). Local delivery of siRNA using a biodegradable polymer application to enhance BMP-induced bone formation. Biomaterials.

[143] Wang Y, Zhang S, Benoit DSW (2018). Degradable poly (ethylene glycol) (PEG)-based hydrogels for spatiotemporal control of siRNA/nanoparticle delivery. J Control Release.

[144] Zhang Y, Wei L, Miron RJ, Shi B, Bian Z (2016). Bone scaffolds loaded with siRNA Semaphorin4d for the treatment of osteoporosis related bone defects. Sci Rep.

[145] Ball RL, Hajj KA, Vizelman J, Bajaj P, Whitehead KA (2018). Lipid nanoparticle formulations for enhanced co-delivery of siRNA and mRNA. Nano Lett.

[146] Park JS, Yang HN, Woo DG, Jeon SY, Park KH (2012). SOX9 gene plus heparinized TGF-b 3 coated dexamethasone loaded PLGA microspheres for inducement of chondrogenesis of hMSCs. Biomaterials.

[147] Jeon SY, Park JS, Yang HN, Woo DG, Park KH (2012). Co-delivery of SOX9 genes and anti-Cbfa-1 siRNA coated onto PLGA nanoparticles for chondrogenesis of human MSCs. Biomaterials.

[148] Park JS, Yang HN, Jeon SY, Woo DG, Kim MS, Park KH (2012). The use of anti-COX2 siRNA coated onto PLGA nanoparticles loading dexamethasone in the treatment of rheumatoid arthritis. Biomaterials.

[149] Jeon SY, Park JS, Yang HN, Lim HJ, Yi SW, Park H (2014). Co-delivery of Cbfa-1-targeting siRNA and SOX9 protein using PLGA nanoparticles to induce chondrogenesis of human mesenchymal stem cells. Biomaterials.

[150] Park JS, Yi SW, Kim HJ, Kim SM, Park KH (2016). Regulation of cell signaling factors using PLGA nanoparticles coated/loaded with genes and proteins for osteogenesis of human mesenchymal stem cells. ACS Appl Mater Interfaces.

[151] Cui ZK, Sun JA, Baljon JJ, Fan J, Kim S, Wu BM (2017). Simultaneous delivery of hydrophobic small molecules and siRNA using Sterosomes to direct mesenchymal stem cell differentiation for bone repair. Acta Biomater.

[152] Li J, Lee WY, Wu T, Xu J, Zhang K, Li G (2016). Multifunctional quantum dot nanoparticles for effective differentiation and long-term tracking of human mesenchymal stem cells in vitro and in vivo. Adv Healthc Mater.

[153] Huang J, Lin C, Fang J, Li X, Wang J, Deng S (2018). pH-sensitive nanocarrier-mediated codelivery of simvastatin and noggin siRNA for synergistic enhancement of osteogenesis. ACS Appl Mater Interfaces.

[154] Bonadio J, Smiley E, Patil P, Goldstein S (1999). Localized, direct plasmid gene delivery in vivo: prolonged therapy results in reproducible tissue regeneration. Nat Med.

[155] Monaghan M, Pandit A (2011). RNA interference therapy via functionalized scaffolds. Adv Drug Deliv Rev.

[156] Sullenger BA, Nair S (2016). From the RNA world to the clinic. Science.

[157] Winkler T, Sass FA, Duda GN, Schmidt-Bleek K (2018). A review of biomaterials in bone defect healing, remaining shortcomings and future opportunities for bone tissue engineering. Bone Joint Res.

[158] Hu Q, Liu M, Chen G, Xu Z, Lv Y (2018). Demineralized bone scaffolds with tunable matrix stiffness for efficient bone integration. ACS Appl Mater Interfaces.

[159] Florentsen CD, West AV, Danielsen HMD, Semsey S, Bendix PM, Oddershede LB (2018). Quantification of loading and laser-assisted release of RNA from single gold nanoparticles. Langmuir.

